# Osmotic activation of a Ca^2+^‐dependent phospholipase C pathway that regulates ∆N TRPV1‐mediated currents in rat supraoptic neurons

**DOI:** 10.14814/phy2.13259

**Published:** 2017-04-21

**Authors:** Vimal Bansal, Thomas E. Fisher

**Affiliations:** ^1^ Department of Physiology, College of MedicineUniversity of SaskatchewanSaskatoonSaskatchewanCanada

**Keywords:** Osmosensitivity, phospholipase C, supraoptic nucleus

## Abstract

The magnocellular neurosecretory cells (MNCs) of the hypothalamus regulate body fluid balance by releasing the hormones vasopressin (VP) and oxytocin (OT) in an osmolality‐dependent manner. Elevations of external osmolality increase MNC firing and hormone release. MNC osmosensitivity is largely due to activation of a mechanosensitive non‐selective cation current that responds to osmotically‐evoked changes in MNC volume and is mediated by an N‐terminal variant of the TRPV1 channel (∆N TRPV1). We report a novel mechanism by which increases in osmolality may modulate ∆N TRPV1‐mediated currents and thus influence MNC electrical behaviour. We showed previously that acute elevations of external osmolality activate the enzyme phospholipase C (PLC) in isolated MNCs. We now show that the osmotic activation of PLC has a time course and dose‐dependence that is consistent with a role in MNC osmosensitivity and that it contributes to the osmotically‐evoked increase in non‐selective cation current in MNCs through a protein kinase C‐dependent pathway. We furthermore show that the mechanism of osmotic activation of PLC requires an increase in internal Ca^2+^ that depends on influx through L‐type Ca^2+^ channels. Our data therefore suggest that MNCs possess an osmotically‐activated Ca^2+^‐dependent PLC that contributes to the osmotic activation of ∆N TRPV1 and may therefore be important in MNC osmosensitivity and in central osmoregulation.

## Introduction

The magnocellular neurosecretory cells of the hypothalamus (MNCs) play a critical role in the regulation of the osmolality of body fluids. When the osmolality of the blood plasma rises above a physiological set‐point, MNCs fire more frequently and release more vasopressin (VP), which decreases urine output and thereby preserves body water, and oxytocin (OT), which in some species complements the effect of VP by increasing the excretion of Na^+^ (Verbalis et al. 1991; Bourque and Oliet 1997). When the plasma has low osmolality, the low concentration of VP enables the production of high volumes of dilute urine to rid the body of excess water. The osmosensitivity of the MNCs depends on osmotically‐induced changes in cell volume (Oliet and Bourque 1993) and the resultant changes in the opening probability of stretch‐inactivated non‐selective cation channels (Oliet and Bourque 1993, 1994). The osmosensitive current is mediated by an N‐terminal variant of the TRPV1 channel (Sharif Naeini et al. 2006) that has been named ∆N TRPV1 (Zaelzer et al. 2015). Activation of these channels depolarizes the MNCs and makes them more likely to fire action potentials in response to excitatory inputs from osmosensitive neurons in neighbouring regions (Oliet and Bourque 1994).

We recently reported that MNCs acutely isolated from the rat supraoptic nucleus (SON) show decreased plasma membrane immunoreactivity to phosphatidylinositol 4, 5‐bisphosphate (PIP_2_) when exposed to increases in external osmolality and that this effect depends on the activation of phospholipase C (PLC; Shah et al. 2014). PLC catalyzes the hydrolysis of PIP_2_ into two other second messengers – diacylglycerol (DAG), which activates protein kinase C, and inositol 1, 4, 5‐trisphosphate (IP_3_), which stimulates the release of Ca^2+^ from internal stores (Rhee 2001). The osmotic activation of PLC could be an important contributor to MNC osmosensitivity because all three of these molecules regulate a variety of cell processes (Suh et al. 2008) and in particular the function of many types of ion channel (Hilgemann et al. 2001). PLC activation has been shown to modulate the activity of TRPV1 channels (Rohacs et al. 2008) and M‐type K^+^ channels (Li et al. 2005), which have also been identified in the MNCs (Zhang et al. 2009).

We therefore sought to characterize the osmotic activation of PLC, determine the mechanism by which it is activated, and determine whether the activation of PLC regulates the activity of ∆N TRPV1 currents in MNCs. We report that the osmotic activation of PLC in MNCs is rapid (occurring within 2 min), reversible, and dose‐dependent, with the maximal effect requiring an approximately 10% increase in osmolality. These observations are consistent with a role for the osmotic activation of PLC in the rapid increases in MNC firing observed in response to sudden increases in external osmolality. We show that osmotic activation of PLC is prevented when Ca^2+^ is absent from the external solution and can be mimicked by administration of a Ca^2+^ ionophore or by high K^+^‐mediated depolarization in the presence of external Ca^2+^. PLC activation is prevented by antagonists of TRPV1 channels or of L‐type Ca^2+^ channels. These data suggest that rat MNCs express a Ca^2+^‐dependent PLC pathway and that this pathway is triggered by an influx of Ca^2+^ through L‐type Ca^2+^ channels that is evoked by TRPV1‐mediated depolarization. We used whole cell patch clamp to test whether PLC plays a role in the osmotic activation of non‐selective cation current in the MNCs and found that the increase in this current is significantly diminished in the presence of a PLC inhibitor. Inclusion of a PIP_2_ analogue in the patch pipette did not alter the response to increases in osmolality, suggesting that the effect does not depend on the observed decrease in PIP_2_, but rather may depend on a product of PIP_2_ hydrolysis such as diacylglycerol (DAG). This hypothesis is supported by experiments showing that inhibition of PKC suppresses the osmotic activation of the non‐selective cation current and that treatment with an agent that mimics DAG potentiates the osmotic activation of this current and directly increases the current when administered in isotonic solution. Our data therefore suggest that MNCs possess an osmotically‐activated PLC pathway that is triggered by Ca^2+^ influx through L‐type Ca^2+^ channels and that modulates the ∆N TPRV1‐mediated current in these cells. The osmotic activation of PLC may play an important role in MNC osmosensitivity and in central osmoregulation in mammals.

## Materials and Methods

### Ethical approval

This work was approved by the University of Saskatchewan's Animal Research Ethics Board and adhered to the Canadian Council on Animal Care guidelines for humane animal use.

### Chemicals

All drugs and chemicals used in the following experiments were purchased from Sigma (St Louis, MO) unless stated otherwise. The PLC inhibitor U73122, the TRPV1 antagonist SB366791, the PKC inhibitor GF‐109203 X, and the inactive analogue of the PKC inhibitor were purchased from Enzo Life Sciences, Inc. (Farmingdale, NY). The PIP_2_ analogue PI(4,5)P_2_‐diC8 was purchased from Echelon Biosciences Inc. (Salt Lake City, UT).

### Animals and cell preparation

Rat MNCs were isolated using a protocol described previously (Liu et al. 2005) and were presumed to be MNCs if they had a maximal cross‐sectional area greater than 160 *μ*m^2^ (Oliet and Bourque 1992). In brief, male Long–Evans rats (200–300 g) were anaesthetized with isoflurane and killed by decapitation. The brain was rapidly removed and blocks of tissue containing most of the two SON were carefully excised. The tissue blocks were then incubated with an oxygenated (100% O_2_) Pipes solution (pH 7.1) composed of (in mmol/L): 120 NaCl, 5 KCl, 1 MgCl_2_, 1 CaCl_2_, 20 Pipes, 10 glucose and containing trypsin (Type XI, 0.6 mg mL^−1^) for 90 min at 34°C. After enzymatic treatment with trypsin, the tissue blocks were transferred into oxygenated Pipes solution (pH 7.4) without trypsin for another 30 min at room temperature. Finally, the tissue blocks were gently triturated with fire‐polished pipettes, dispersed onto glass‐bottomed culture dishes and kept at room temperature for electrophysiological or immunocytochemical experiments as discussed below.

### PIP_2_ immunocytochemistry

We pooled MNCs from two or three rats for each immunocytochemical experiment to ensure that we obtained enough cells. Acutely isolated rat MNCs plated on glass‐bottom dishes were perfused with different experimental solutions for specific time periods as indicated in the text. The osmolalities of all solutions were adjusted by adding mannitol and measured using a VAPRO pressure osmometer (WESCOR; Logan, UT). The high K^+^ saline was prepared by iso‐osmotic substitution of 25 mmol/L NaCl with 25 mmol/L KCL saline of the isotonic Pipes solution. At the end of each experiment, the MNCs were subjected to phosphatidylinositol 4, 5‐bisphosphate (PIP_2_) immunostaining using a modification of a published protocol (Hammond et al. 2006) that has been used by us previously (Shah et al. 2014). Briefly, the cells were fixed with phosphate‐buffered saline (PBS) containing 4% paraformaldehyde for 20–25 min at room temperature. Following three washes with PBS, the cells were blocked with solution containing 10% donkey serum and 0.5% saponin for 1 h. The cells were then incubated with a mouse monoclonal PIP_2_ antibody (Enzo Life Sciences; 1:1000; Fukami et al. 1988; Defacque et al. 2002) overnight at 4°C. The next day, dishes were washed with PBS three times and incubated for 1 h with donkey anti‐mouse secondary antibody (Invitrogen Alexa Fluor 488, 1:1000). After three washes with PBS, Citifluor mounting solution (Citifluor Ltd; Gore, QC, Canada) was added to the dishes and cells were then viewed using a Zeiss inverted Axiovert 200 microscope with appropriate filter sets and a 40X objective lens. An attached cooled CCD camera was used to capture images of all cells that were deemed to be MNCs (based on the size criterion mentioned above), appeared to be healthy (had a clear cell perimeter and were not bloated), and were well attached to the dish. All the captured images were later analyzed using Image‐J software (NIH) by tracing the perimeter of each MNC by following the line of greatest fluorescence (disregarding processes) and determining the mean fluorescence of pixels on that line. Negative control experiments in which the primary antibody was excluded showed no significant staining of the MNC plasma membrane. The mean intensities of staining for all MNCs in each treatment group were then normalized to the mean fluorescence of all the control cells done on each experimental day and expressed as normalized mean ± SEM for each group. The normalized intensities of the treatment group or groups were then statistically compared with the control group values using either a student's paired *t* test or a repeated measures one‐way ANOVA followed by a Dunnett's multiple comparison test when applicable. The differences were deemed significant if *P* < 0.05.

### Electrophysiological methods

The plated MNCs were maintained in an isotonic (295 mosmol kg^−1^) external recording solution having (in mmol/L): 140 NaCl, 5 KCl, 10 Hepes, 1 MgCl_2_, 1 CaCl_2_, 10 glucose and 0.0005 tetrodotoxin (pH 7.4) and placed on the microscope stage of the electrophysiology station for current measurements using an EPC‐9 amplifier (HEKA Elektronik; Lambrecht/Pfalz, Germany) controlled with PULSE software (HEKA). The whole cell patch clamp configuration was achieved using patch pipettes having resistance of 2–4 MΩ and filled with an internal solution (osmolality 280 mosmol kg^−1^ and pH 7.2) having (in mmol/L): 125 KCl, 10 Hepes, 1 MgCl_2_, 0.5 EGTA, 4 Na_2_‐ATP, 1 Na‐GTP, and 14 phosphocreatine unless otherwise stated. The osmolalities of all perfusing solutions were adjusted by adding mannitol. Borosilicate glass capillaries (1.2 mm o.d., 0.68 mm i.d; A‐M Systems; Carlsborg, WA) were used to pull patch pipettes on a P‐97 horizontal pipette puller (Sutter Instrument Company; Novato) and fire‐polished using a microforge (Narashige; Tokyo, Japan). The MNCS were exposed to different experimental treatment conditions as specified in the result section and macroscopic whole cell ramp currents were evoked from a holding potential of −70 mV using a 5 sec voltage ramp protocol from −100 mV to −20 mV under isotonic conditions. The cells were then switched to current clamp mode and perfused with hypertonic saline solution before re‐recording the macroscopic whole cell ramp currents to estimate the effects of different experimental treatments. All current measurements were made using PULSEFIT software (HEKA) and the signals were low‐pass filtered at 2 kHz and digitized at 20 kHz for all experiments. The individual current traces obtained in isotonic and hypertonic conditions for all the MNCs belonging to a particular treatment group were averaged to generate mean ramp current traces before and after treatment. These traces were then digitally subtracted to obtain the current evoked by each treatment. The mean reversal potentials of the evoked currents were calculated by averaging the individual reversal potentials and were expressed as mean ± SEM. The peak amplitude of the osmotically‐evoked ramp current (mean current between −100 and −90 mV at the beginning of the ramp protocol) of all MNCs was also measured and normalized by dividing the current value by the whole cell capacitance to obtain a peak osmosensitive current density. These individual peak current densities were then pooled and averaged for all treatment groups and expressed as mean ± SEM. We also calculated the osmotically‐induced increase in membrane conductance of all MNCs by measuring the difference in the slope values of their *I*–*V* plots between −100 and −50 mV (the linear region) obtained under isotonic and hypertonic conditions. Increases in conductance were pooled and expressed as mean ± SEM. The mean reversal potentials, averaged peak osmosensitive current density, and mean increase in membrane conductance values for each experimental group were statistically compared with the respective control group values, as detailed in the results, using a student's unpaired *t* test. The differences were deemed significant if *P* < 0.05.

## Results

In our previous paper (Shah et al. 2014) we reported that acute exposure of isolated MNCs to increases in external osmolality activates PLC resulting in a decrease in plasma membrane immunoreactivity to PIP_2_. We presented evidence that this activation may play a role in osmotically‐evoked hypertrophy of MNCs, which has been observed in vivo (Hatton 1997) and that we demonstrated in isolated MNCs following exposure to hypertonic solutions lasting tens of minutes (Shah et al. 2014). We hypothesized in that paper that activation of PLC might also play a role in the modulation of ion channels that mediates the intrinsic osmosensitivity of MNCs, but did not test this hypothesis. Our first aim was therefore to test whether the extent, time course, and osmotic dependence of PLC activation are consistent with the electrophysiological changes that occur in response to increases in external osmolality.

### Activation of PLC by hypertonic external solutions, by receptor‐mediated stimulation with angiotensin II, and by direct stimulation with a PLC activator all decrease membrane PIP_2_ to a similar extent

**Figure 1:**
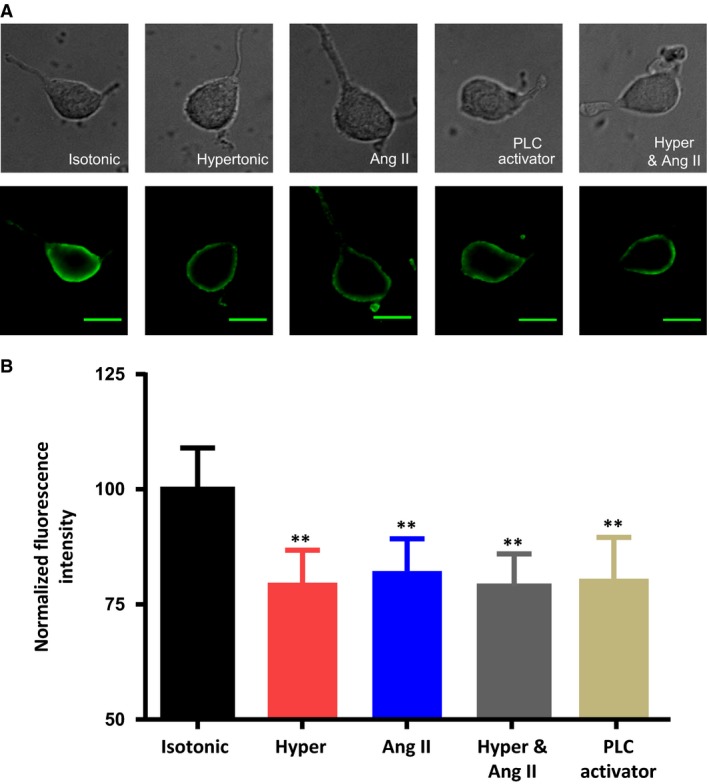
Exposure of acutely isolated rat MNCs to hypertonic saline, Ang II, hypertonic saline with Ang II, or a PLC activator all decrease membrane PIP_2_ to a similar extent. (A) images of isolated MNCs using either differential interference contrast images (upper panels) or fluorescence images showing immunoreactivity for PIP_2_ (lower panels) maintained in isotonic saline or exposed to hypertonic saline (325 mosmol kg^−1^), Ang II (5 *μ*mol/L), hypertonic saline containing Ang II (5 *μ*mol/L), or the PLC activator 3M3FBS (30 *μ*mol/L) for 5 min. The scale bar indicates 20 *μ*m. (B) The bar graph shows the mean normalized immunoreactivity to PIP_2_ in the five conditions. Data are expressed as mean normalized fluorescence intensity ± SEM (*P < *0.01 is indicated by **).

We compared the magnitude of the osmotically‐evoked decrease in PIP_2_ immunoreactivity to the decreases caused by other means of PLC stimulation to determine whether the osmotically‐induced decrease in PIP_2_ is consistent with a physiologically‐important activation of PLC. Figure 1 compares the effects on PIP_2_ immunoreactivity caused by exposure to hypertonic saline (325 mosmol kg^−1^), to angiotensin II (Ang II; 5 *μ*mol/L), to hypertonic saline with Ang II, and to the non‐specific PLC activator m‐3M3FBS (30 *μ*mol/L). Angiotensin II (Ang II) was chosen because it activates the PLC pathway in rat MNCs through a receptor‐dependent mechanism (Chakfe and Bourque 2000; Zhang and Bourque, 2008) and 5 *μ*mol/L Ang II was chosen because that dose would be expected to cause maximal activation of the receptor‐linked PLC pathway (Chakfe and Bourque 2000, 2001). As illustrated in Figure 1, exposure to each of these treatments for 5 min caused a similar decrease in PIP_2_ immunoreactivity (about 20% in each case). These results are summarized in the bar graphs in Figure 1B. Our results suggest that the osmotically‐evoked decrease in PIP_2_ is likely to be physiologically significant and that osmotic activation of PLC may contribute to the intrinsic osmosensitivity of the MNCs in ways that overlap with the effects caused by Ang II (Chakfe and Bourque 2000; Zhang and Bourque 2008).

### Acute exposure to hypertonic saline causes a reversible, time‐ and dose‐dependent decrease in PIP_2_ immunoreactivity

**Figure 2:**
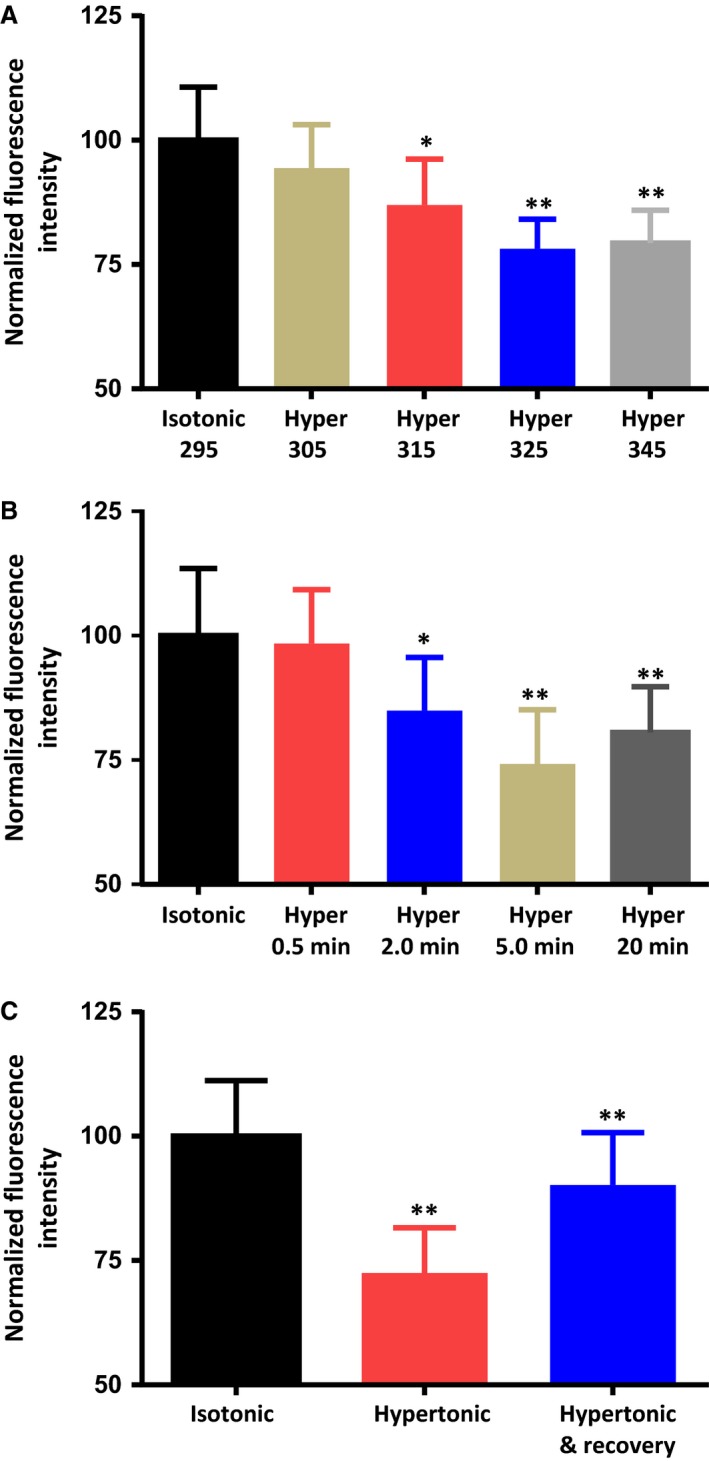
Exposure to hypertonic saline causes a dose‐dependent, time‐dependent, reversible decrease in immunoreactivity to PIP_2_ in the plasma membrane of isolated MNCs. (A) The bar graph shows the mean normalized immunoreactivity to PIP_2_ in MNCs maintained in isotonic saline or hypertonic saline of 305, 315, 325, and 345 mosmol kg^−1^ for 5 min. (B) The bar graph shows the mean normalized immunoreactivity to PIP_2_ in MNCs maintained in isotonic saline or exposed to hypertonic saline (325 mosmol kg^−1^) for the indicated times. (C) The bar graph shows the mean normalized immunoreactivity to PIP_2_ in MNCs maintained in isotonic saline, after 5 min in hypertonic saline (325 mosmol kg^−1^), and after 5 min in hypertonic saline and 5 min in isotonic saline. The values for the “hypertonic & recovery” MNCs were compared to those of the “hypertonic” MNCs. Data are expressed as mean normalized fluorescence intensity ± SEM (*P < *0.05 is indicated by *; *P < *0.01 by **).

We therefore tested whether the dose and time dependence of the osmotically‐evoked activation of PLC are consistent with the intrinsic osmosensitive properties of the MNCs. We perfused dishes of isolated MNCs with saline solutions of five different osmolalities (305, 315, 325 and 345 mosmol kg^−1^) for 5 min and then compared their mean PIP_2_ immunoreactivity to that of the control MNCs that were incubated in isotonic saline (295 mosmol kg^−1^). As illustrated in Figure 2A, the decrease in membrane PIP_2_ was found to occur in a dose‐dependent fashion and was statistically significant at all osmolalities equal to or higher than 315 mosmol kg^−1^. The response appeared to be maximal at an osmolality of 325 mosmol kg^−1^ and was not larger at an osmolality of 345 mosmol kg^−1^. The normalized PIP_2_ immunoreactivity observed during these experiments were as follows: 295 mosmol kg^−1^ (control; 100.0 ± 10.7; *n *=* *174 cells in 5 experiments), 305 mosmol kg^−1^ (94.4 ± 8.7; *n *=* *166 cells in 5 experiments), 315 mosmol kg^−1^ (87.0 ± 9.2; *n *=* *155 cells in 5 experiment), 325 mosmol kg^−1^ (78.2 ± 5.9; *n *=* *185 cells in 5 experiments) and 345 mosmol kg^−1^ (79.3 ± 6.3; *n *=* *161 cells in 5 experiments). Data are expressed as mean normalized fluorescence intensity ± SEM (*P < *0.05; *P < *0.01).

We next sought to determine the time course of the response. We exposed dishes of MNCs to 325 mosmol kg^−1^ saline solutions for four different periods (30 sec, 2 min, 5 min and 20 min) and compared their mean membrane fluorescence values with those of the control cells that were incubated in isotonic solution. As illustrated in Figure 2B, a statistically‐significant decrease in membrane PIP_2_ was observed at 2 min, was maximal at 5 min, and persisted for 20 min with continued exposure to hypertonic saline. The mean normalized PIP_2_ immunoreactivity values observed were as follows: isotonic (control; 100.0 ± 13.5; *n *=* *234 cells in 6 experiments), 30 sec exposure (98.4 ± 10.8; *n *=* *155 cells in 6 experiments), 2‐min exposure (84.9 ± 10.7; *n *=* *177 cells in 6 experiments), 5‐min exposure (74.2 ± 10.9; *n *=* *222 cells in 6 experiments) and 20‐min exposure (80.6 ± 9.2; *n *=* *188 cells in 6 experiments) to hypertonic saline (325 mosmol kg^−1^).

We next tested if the osmotically‐evoked decrease in PIP_2_ could be reversed by re‐exposure to isotonic saline solution. We perfused dishes of MNCs with 325 mosmol kg^−1^ saline for 5 min and then replaced the solution with 295 mosmol kg^−1^ solution for another 5 min. As illustrated in Figure 2C, PIP_2_ immunoreactivity showed a significant recovery toward control levels after a 5 min exposure to isotonic saline. The normalized PIP_2_ immunoreactivity values were as follows: isotonic (control; 100.0 ± 11.2; *n *=* *228 cells in 6 experiments), hypertonic for 5 min (72.5 ± 9.1; *n *=* *161 cells in 6 experiments) and hypertonic for 5 min followed by a 5‐min isotonic re‐exposure (90.2 ± 10.6; *n *=* *189 cells in 6 experiments). The rapid activation and reversibility of the osmotically‐evoked PLC effect is consistent with a role for this effect in the intrinsic osmosensitivity of the MNCs.

### The osmotically‐evoked decrease in membrane PIP_2_ is dependent on the osmotic activation of ∆N TRPV1 channels and on the influx of extracellular Ca^2+^ through L‐type Ca^2+^ channels

**Figure 3:**
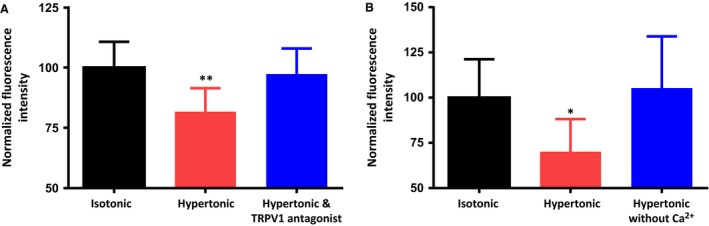
The osmotically‐evoked decrease in membrane PIP_2_ depends on the activation of TRPV1 channels and on extracellular Ca^2+^. (A) The bar graph shows the mean normalized immunoreactivity to PIP_2_ in MNCs maintained in isotonic saline, exposed to hypertonic (325 mosmol kg^−1^) saline for 5 min, and exposed to hypertonic saline in the presence of the TRPV1 channel antagonist SB366791 (5 *μ*mol/L) for 5 min. (B) The bar graph shows the mean normalized immunoreactivity to PIP_2_ in MNCs maintained in isotonic saline, exposed to hypertonic (325 mosmol kg^−1^) saline for 5 min, and exposed to hypertonic saline that contains no added Ca^2+^ for 5 min. Data are expressed as mean normalized fluorescence intensity ± SEM (*P < *0.05 is indicated by *; *P < *0.01 by **).

As mentioned above, the osmotically‐evoked excitation of MNCs depends on the activation of ∆N TRPV1 channels (Sharif Naeini et al. 2006; Zaelzer et al. 2015). We therefore exposed dishes of MNCs to either 325 mosmol kg^−1^ saline containing vehicle (DMSO) or 325 mosmol kg^−1^ saline containing 5 *μ*mol/L of the specific TRPV1 channel antagonist SB366791 (which blocks TRPV1 channels in MNCs; Sharif‐Naeini et al. 2008; Sudbury and Bourque 2013). As illustrated in Figure 3A, the osmotically‐evoked decrease in membrane PIP_2_ was effectively prevented by SB366791, which suggests that PLC is not directly activated by increases in osmolality, but rather requires the activation of ∆N TRPV1 channels. The mean normalized PIP_2_ immunoreactivity values observed during these experiments were as follows: isotonic saline (control; 100.0 ± 10.7; *n *=* *169 cells in 5 experiments), hypertonic saline containing DMSO (81.7 ± 9.7; *n *=* *151 cells in 5 experiments) and hypertonic saline containing SB366791 (97.3 ± 10.6; *n *=* *172 cells in 5 experiments).

Ca^2+^ influx has been shown to activate PLC pathways in other cell types (Lukacs et al. 2013; Borbiro et al. 2015) and we therefore hypothesized that Ca^2+^ influx could be the trigger for the osmotically‐evoked activation of PLC. We therefore treated subsets of MNCs for 5 min with either hypertonic saline (325 mosmol kg^−1^) or hypertonic saline made with no added Ca^2+^. The mean membrane fluorescence values of all cells were then compared with the control cells that were incubated in isotonic saline. As illustrated in Figure 3B, the osmotically‐evoked decrease in membrane PIP_2_ was effectively eliminated in the absence of external Ca^2+^. The mean normalized PIP_2_ immunoreactivity values in these experiments were as follows: isotonic saline (control; 100.0 ± 21.2; *n *=* *205 cells in 5 experiments), hypertonic saline (70.2 ± 18; *n *=* *198 cells in 5 experiments) and hypertonic saline with no added Ca^2+^ (105.3 ± 28.54; *n *=* *147 cells in 5 experiments). These results suggest that osmotic activation of PLC is a Ca^2+^ dependent process.

### The osmotically‐evoked activation of PLC depends on Ca^2+^ influx through L‐type Ca^2+^ channels

**Figure 4:**
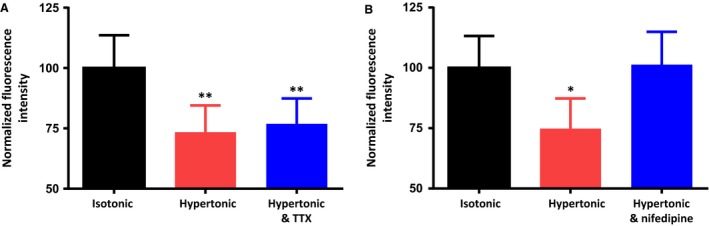
The osmotically‐evoked decrease in membrane PIP_2_ does not depend on action potential firing, but does depend on Ca^2+^ influx through L‐type Ca^2+^ channels. (A) The bar graph shows the mean normalized immunoreactivity to PIP_2_ in MNCs maintained in isotonic saline, exposed to hypertonic saline (325 mosmol kg^−1^) for 5 min, and exposed to hypertonic saline (325 mosmol kg^−1^) for 5 min in the presence of the Na^+^ channel antagonist tetrodotoxin (TTX; 0.5 *μ*mol/L). (B) The bar graph shows the mean normalized immunoreactivity to PIP_2_ in MNCs maintained in isotonic saline, exposed to hypertonic saline (325 mosmol kg^−1^) for 5 min, and exposed to hypertonic saline (325 mosmol kg^−1^) for 5 min in the presence of the L‐type Ca^2+^ channel antagonist nifedipine (30 *μ*mol/L). Data are expressed as mean normalized fluorescence intensity ± SEM (*P < *0.05 is indicated by *; *P < *0.01 by **).

**Figure 5:**
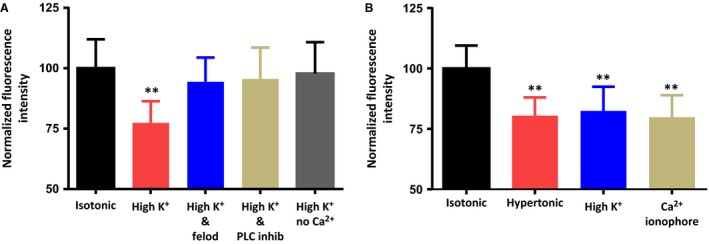
The osmotically‐evoked decrease in membrane PIP_2_ can be mimicked by high K^+^‐induced Ca^2+^ influx though L –type Ca^2+^ channels or by a Ca^2+^ ionophore. (A) The bar graph shows the mean normalized immunoreactivity to PIP_2_ in MNCs maintained in isotonic saline, exposed to high K^+^ (30 mmol/L) saline for 5 min, exposed to high K^+^ saline in the presence of the L‐type Ca^2+^ channel antagonist felodipine (30 *μ*mol/L) for 5 min, exposed to high K^+^ saline in the presence of the PLC inhibitor U‐73122 (1 *μ*mol/L) for 5 min, and exposed to high K^+^ saline that contains no added Ca^2+^ for 5 min. (B) The bar graph shows the mean normalized immunoreactivity to PIP_2_ in MNCs maintained in isotonic saline, exposed to hypertonic saline (325 mosmol kg^−1^) for 5 min, exposed to high K^+^ saline for 5 min, and exposed to the Ca^2+^ ionophore A23187 (20 *μ*mol/L) for 5 min. Data are expressed as mean normalized fluorescence intensity ± SEM (*P < *0.01 is indicated by **).

Osmotically‐evoked Ca^2+^ influx could occur through the ∆N TRPV1 channels themselves, which are Ca^2+^ permeable (Zhang and Bourque 2006), or it could depend on ∆N TRPV1‐mediated depolarization and the activation of voltage‐dependent Ca^2+^ channels, several of which are expressed in MNCs (Fisher and Bourque 1995; Foehring and Armstrong 1996). We used the Na^+^ channel antagonist tetrodotoxin (TTX) to test whether the response requires the firing of action potentials. Figure 4A shows that the inclusion of 0.5 *μ*mol/L TTX during a 5‐min hypertonic treatment did not significantly alter the osmotically‐induced reduction in PIP_2_, suggesting that under our conditions the firing of action potentials is not necessary to activate this response. The normalized PIP_2_ immunoreactivity values observed were as follows: isotonic saline (control; 100.0 ± 13.6 *n *=* *172 cells in 5 experiments), hypertonic (325 mosmol kg^−1^) saline (73.4 ± 11.1; *n *=* *176 cells in 5 experiments), and hypertonic saline in the presence of 0.5 *μ*mol/L TTX (76.9 ± 10.5; *n *=* *147 cells in 5 experiments). The inclusion of 30 *μ*mol/L of the L‐type Ca^2+^ channel antagonist nifedipine, however, effectively blocked the response (Fig. 4B), suggesting that Ca^2+^ influx through L‐type Ca^2+^ channels is required. The normalized PIP_2_ immunoreactivity values observed during these experiments were as follows: isotonic saline (control; 100.0 ± 13.2 *n* = 228 cells in 6 experiments), hypertonic saline containing DMSO (74.9 ± 12.5; *n* = 243 cells in 6 experiments), and hypertonic saline containing 30 *μ*mol/L nifedipine (101.3 ± 13.6; *n* = 160 cells in 6 experiments). Figure 5A shows that exposure of the MNCs to an isotonic high K^+^ (30 mmol/L) saline (prepared by iso‐osmotic substitution of 25 mmol/L NaCl with 25 mmol/L KCl) causes a decrease in membrane PIP_2_ and that the decrease is prevented by the L‐type Ca^2+^ channel antagonist felodipine (30 *μ*mol/L) or the PLC inhibitor U‐73122 (1 *μ*mol/L) or by removal of Ca^2+^ from the external solution. Note that in the high K^+^ experiments, 0.5 *μ*mol/L TTX was also added to ensure that the cells were not firing action potentials. The mean normalized PIP_2_ immunoreactivity values observed during these experiments were as follows: isotonic saline (control; 100.0 ± 11.6; *n* = 156 cells in 5 experiments), high K^+^ isotonic saline (77.4 ± 9.5; *n* = 168 cells in 5 experiments), high K^+^ isotonic saline in the presence of 30 *μ*mol/L felodipine (94.4 ± 9.7; *n* = 150 cells in 5 experiments), high K^+^ isotonic saline following a pre‐treatment with U‐73122 (95.5 ± 12.8; *n* = 141 cells in 5 experiment), and high K^+^ saline with no added Ca^2+^ (97.8 ± 13.2; *n* = 108 cells in 5 experiments). These experiments demonstrate that PLC can be activated by high K^+^‐induced depolarization and the resultant influx of Ca^2+^ through L‐type Ca^2+^ channels. These data also suggest that influx of Ca^2+^ through ∆N TRPV1 channels is not necessary for the activation of PLC, but the influx through L‐type Ca^2+^ channels is required whether cell depolarization is mediated by the activation of ∆N TRPV1 channels or by an increase in the concentration of external K^+^. Figure 5B shows that treatment with the Ca^2+^ ionophore A23187 (20 *μ*mol/L) causes a decrease in PIP_2_ immunoreactivity that is similar to that observed with either hypertonic treatment or exposure to high K^+^ saline. The normalized PIP_2_ immunoreactivity values observed during these experiments were as follows: isotonic saline (control; 100.0 ± 9.5; *n* = 183 cells in 5 experiments), hypertonic (325 mosmol kg^−1^) saline (80.5 ± 7.5; *n* = 152 cells in 5 experiments), high K^+^ isotonic saline (82.4 ± 10.0; *n* = 170 cells in 5 experiments), and isotonic saline containing 20 *μ*mol/L A23187 (79.9 ± 9.1; *n* = 137 cells in 5 experiments). These data support the hypothesis that Ca^2+^ influx is necessary and sufficient to activate PLC.

### Osmotic activation of PLC contributes to the osmotic activation of ∆N TRPV1 currents

**Figure 6:**
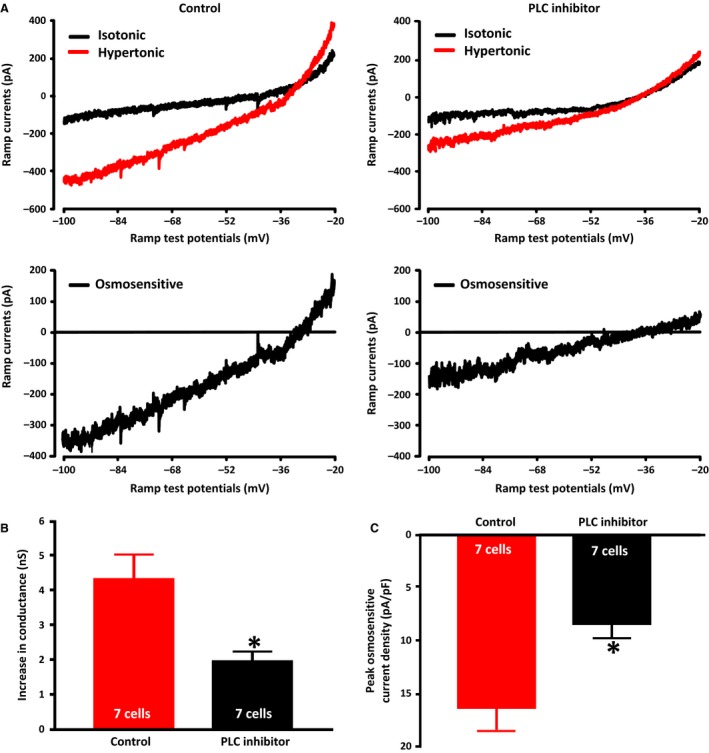
PLC Inhibition reduces osmotically‐evoked TRPV1 currents. (A) The upper panels show the mean ramp current traces evoked before (black trace) and after (red trace) exposure to hypertonic (345 mosmol kg^−1^) in the absence (left traces) and presence (right traces) of the PLC inhibitor U73122 (1 *μ*mol/L). The lower traces show the digital subtraction of the black and red traces, which therefore represents the osmotically‐evoked current under the two conditions. (B) The bar graphs show the mean osmotically‐evoked increase in the membrane conductance of MNCs in the two conditions. (C) The bar graphs show the mean peak densities of osmotically‐evoked currents in the two conditions. Data are expressed as mean ± SEM (*P < *0.05 is indicated by *).

MNC excitability is enhanced by treatment with muscarine (Ghamari‐Langroudi and Bourque 2004) or Ang II (Yang et al. 1992; Chakfe and Bourque 2000, 2001), both of which act at G‐protein coupled receptors that lead to the activation of PLC (Oude Weernink et al. 2007). Although the mechanism by which muscarine increases excitability is not clear (Ghamari‐Langroudi and Bourque 2004), Ang II has been shown to mediate its stimulatory effect by increasing osmotically‐evoked non‐selective cation currents in a PLC‐dependent fashion (Chakfe and Bourque 2000, 2000, 2001; Zhang and Bourque 2008) that was proposed to involve cytoskeleton‐dependent changes in transducer gain (Chakfe and Bourque 2000; Zhang and Bourque 2008). We therefore hypothesized that osmotic activation of PLC could contribute to the regulation of osmotically‐regulated non‐selective cation channels, which are now known to be mediated by ∆N TRPV1 channels (Sharif Naeini et al. 2006; Zaelzer et al. 2015). We tested this possibility using whole cell patch clamp techniques on MNCs to measure ramp currents (from −100 mV to −20 mV) before and after exposure to hypertonic solutions following pretreatment with either vehicle (DMSO) or the PLC inhibitor U‐73122 (1 *μ*mol/L) for 20 minutes. After stable ramp currents were obtained, the isotonic saline was replaced with hypertonic (345 mosmol kg^−1^) saline and the ramp currents were recorded again. The traces in the upper part of Figure 6A are the means of the ramp currents for cells before and after the addition of hypertonic saline. The traces in the lower panels are the result of digital subtraction of the two currents and therefore show the osmotically‐evoked currents in the cells exposed to hypertonic saline. The increase in osmolality activated a current with a mean reversal potential of about −30 mV (Fig. 6A), which is consistent with what has been shown for osmotically‐evoked ramp currents in MNCs (Voisin et al. 1999; Zhang et al. 2007b). Addition of SB‐366791 was able to reverse the osmotically‐evoked increase in ramp currents (*n* = 2) confirming that the osmotically evoked ramp currents are indeed mediated by TRPV1 channels (data not shown). The reversal potentials in the vehicle control group (−29.9 ± 2.3 mV) and the PLC inhibitor group (−34.0 ± 3.1 mV) were not statistically different. Figure 6 however shows that the osmotically‐evoked currents were significantly diminished in the presence of the PLC inhibitor U73122 (1 *μ*mol/L), both in terms of the increase in conductance and in the peak current density of the osmotically‐evoked current. Figure 6B shows that the mean increase in membrane conductance in the PLC inhibitor group (1.98 ± 0.25 nS; *n *=* *7 cells) was significantly lower than in the control group (4.34 ± 0.68 nS; *n *=* *7 cells) and Figure 6C shows that the average peak ramp current density in the PLC inhibitor group (8.5 ± 1.2 pA/pF; *n *=* *7 cells) was significantly lower than in the control group (16.3 ± 2.1 pA/pF; *n *=* *7 cells). These data suggest that PLC contributes to the activation of the ∆N TRPV1‐mediated current that occurs in response to elevations in external osmolality.

### The PLC‐mediated enhancement of the ∆N TRPV1‐mediated current depends on the activation of PKC

**Figure 7:**
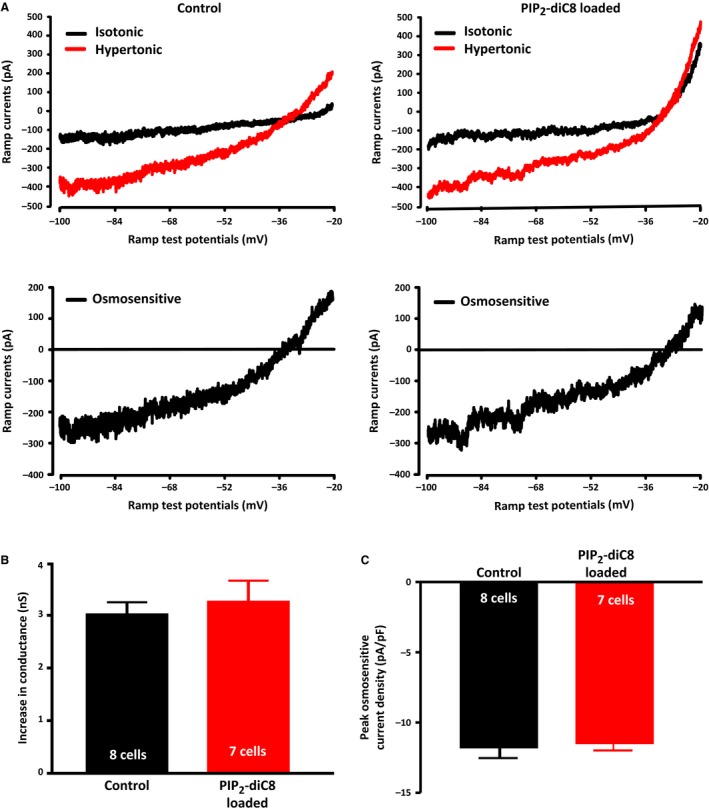
A PIP_2_ analogue does not affect the osmotic activation of TRPV1 currents. (A) The upper panels show the mean ramp current traces evoked before (black trace) and after (red trace) exposure to hypertonic (325 mosmol kg^−1^) in the absence (left traces) and presence (right traces) of the PIP_2_ analogue PIP_2_‐diC8 (100 *μ*mol/L) in the patch pipette. The lower traces show the digital subtraction of the black and red traces, which therefore represents the osmotically‐evoked current under the two conditions. (B) The bar graphs show the mean osmotically‐evoked increase in the membrane conductance of MNCs in the two conditions. (C) The bar graphs show the mean peak densities of osmotically‐evoked currents in the two conditions. Data are expressed as mean ± SEM.

The observation that inhibition of PLC results in a decrease in the osmotic activation of the ∆N TRPV1‐mediated current suggests that ∆N TRPV1 channels are either inhibited by PIP_2_ (and thus are enhanced by a decrease in PIP_2_) or are activated by one or both of the downstream products of PLC (IP_3_ and DAG). We therefore tested whether inclusion of the water‐soluble PIP_2_ analogue PIP_2_‐diC8 (100 *μ*mol/L) in the recording patch pipette would inhibit the osmotic activation of the ∆N TRPV1‐mediated current. PIP_2_‐diC8 has been shown to mimic the effects of PIP_2_ on ion channels in other cell types (Lukacs et al. 2007; Albert et al. 2008) and its presence should compensate for any evoked decreases in PIP_2_ concentration. The average ramp current traces obtained under isotonic and hypertonic (325 mosmol kg^−1^) conditions for both control and PIP_2_‐diC8‐loaded MNCs are shown in the top panels of Figure 7A and the digitally‐subtracted average osmotically‐evoked currents are shown in the bottom panels. The mean reversal potential of the osmotically‐evoked currents in control (−34.1 ± 2.8 mV) and PIP_2_‐diC8‐loaded MNCs (−30.1 ± 4.3 mV) were not statistically different. As illustrated in Figure 7B, the mean peak ramp current densities observed in PIP_2_‐loaded MNCs (11.5 ± 0.4 pA/pF; *n *=* *7 cells) was not significantly different from those of the control MNCs (11.9 ± 0.7 pA/pF; *n *=* *8). The mean increase in membrane conductance of the PIP_2_‐diC8‐loaded MNCs (3.28 ± 0.38 nS; *n *=* *7 cells) was also not significantly different from the control MNCs (3.04 ± 0.21 nS; *n *=* *8 cells), as is shown in Figure 7C. These data suggest that the effect of PLC activation on the ∆N TRPV1‐mediated current does not depend on a decrease in the concentration of PIP_2_.

**Figure 8:**
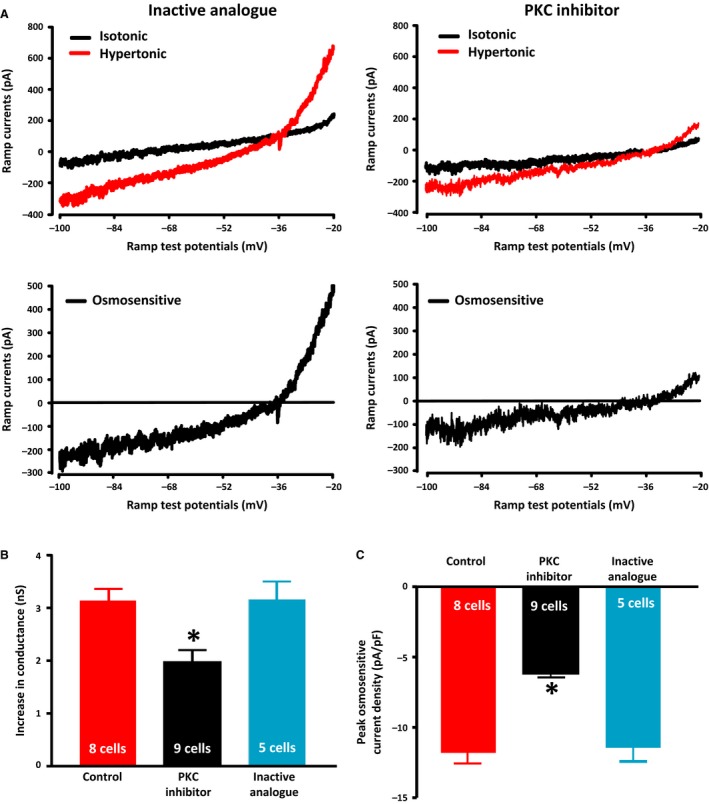
PKC inhibition suppresses the osmotic activation of TRPV1 currents. (A) The upper panels show the mean ramp current traces evoked before (black trace) and after (red trace) exposure to hypertonic (325 mosmol kg^−1^) in the presence of an inactive analogue and the PKC inhibitor GF109203X (2 *μ*mol/L). The lower traces show the digital subtraction of the black and red traces, which therefore represents the osmotically‐evoked current under the two conditions. (B) The bar graphs show the mean osmotically‐evoked increase in the membrane conductance of MNCs under control conditions (data from Fig. 7) or following treatment with PKC inhibitor or inactive analogue. (C) The bar graphs show the mean peak densities of osmotically‐evoked currents in the three conditions. Data are expressed as mean ± SEM (*P < *0.05 is indicated by *).

We then pre‐treated MNCs with the PKC inhibitor GF109203X (2 *μ*mol/L) to test whether PKC activation is involved in the osmotic activation of the ∆N TRPV1‐mediated current. The ramp currents were measured before and after exposure to hypertonic solution as described above and were compared to the currents in cells pre‐treated with 2 *μ*mol/L of the inactive PKC inhibitor analogue bisindolylmaleimide V for 20 minutes. The mean ramp current traces obtained under isotonic and hypertonic (325 mosmol kg^−1^) conditions are shown in the top panels of Figure 8A and the digitally‐subtracted mean osmotically‐evoked currents are shown in the bottom panels. The mean reversal potential of the osmotically‐evoked currents obtained in the presence of the inactive analogue (−33.6 ± 2.4 mV) and the PKC inhibitor (−31.3 ± 2.7 mV) were not statistically different. The mean peak ramp current densities in the PKC inhibitor (6.3 ± 0.3 pA/pF; *n *=* *9 cells) were significantly smaller than those in the inactive analogue (11.4 ± 1.0 pA/pF; *n *=* *5 cells) as well as that found for the control group (11.9 ± 0.7 pA/pF; *n *=* *8) in the previous experiment. The mean increase in membrane conductance in the PKC inhibitor (1.99 ± 0.09 nS; *n *=* *9 cells) was also significantly smaller than in the inactive analogue (3.16 ± 0.34 nS; *n *=* *5 cells) as well as that observed in the control group (3.04 ± 0.21 nS; *n *=* *8 cells) from the last experiment (Fig. 8C). The mean peak ramp current density and the mean increase in cellular conductance observed in the MNCs treated with the inactive PKC analogue were not different than those obtained from the control group in the previous experiment. These data suggest that the activation of PLC contributes to the osmotically‐evoked increase in TRPV1 currents through a PKC‐dependent mechanism.

### A PKC activator increases the basal ∆N TRPV1‐mediated current and also enhances the osmotic activation of ∆N TRPV1‐mediated current

**Figure 9:**
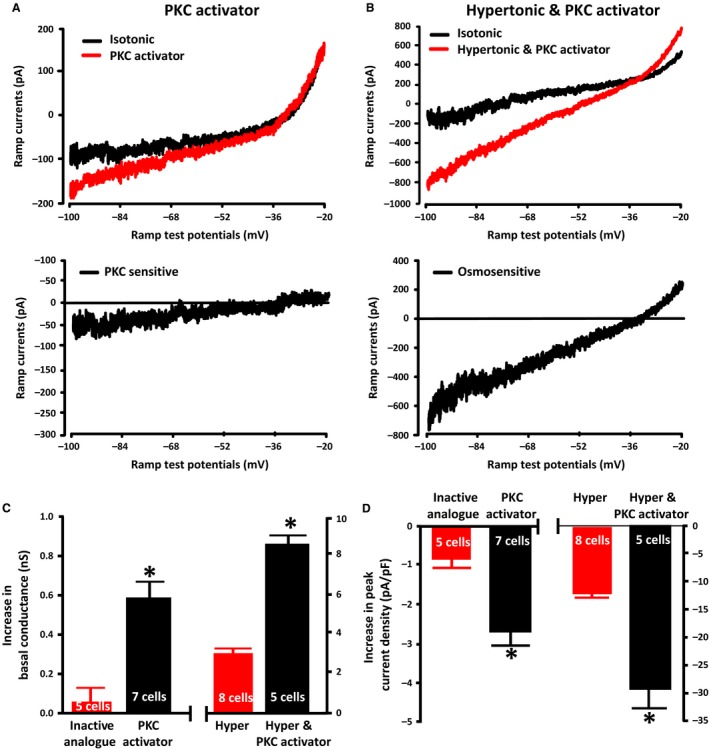
PKC activation increases TRPV1 currents in isotonic saline and enhances the osmotic activation of TRPV1 currents. (A) The upper panel shows the mean ramp current traces evoked before (black trace) and after (red trace) exposure to the PKC activator phorbol 12‐myristate 13‐acetate (PMA; 0.2 *μ*mol/L) in isotonic saline. The lower trace shows the digital subtraction of the black and red traces, which therefore represents the PKC activated current. (B) The upper panels on the right show the mean ramp current traces evoked before (black trace) and after (red trace) exposure to hypertonic saline (325 mosmol kg^−1^) in the presence of the PKC activator phorbol 12‐myristate 13‐acetate (PMA; 0.2 *μ*mol/L). The lower trace shows the digital subtraction of the black and red traces, which therefore represents the osmotically‐evoked current in the presence of PMA. (C) The bar graph on the left shows the mean increase in the membrane conductance of MNCs caused by the PKC activator and the bar graph on the right shows the effect of hypertonic saline (325 mosmol kg^−1^) in the presence of the PKC activator compared to the effect of hypertonic saline (325 mosmol kg^−1^) in the absence of the PKC activator (data from the previous experiment). (D) The bar graph on the left shows the mean membrane conductance of MNCs in the presence of the inactive analogue and the PKC activator and the bar graph on the right shows the effect of hypertonic saline (325 mosmol kg^−1^) in the presence of the PKC activator compared to the effect of hypertonic saline (325 mosmol kg^−1^) in the absence of the PKC activator (data from the previous experiment). Data are expressed as mean ± SEM (*P < *0.05 is indicated by *).

The observation that inhibition of PKC suppresses the osmotic activation of ∆N TRPV1‐mediated current suggests that PKC may regulate ∆N TRPV1 channel activity. We therefore tested whether PKC activation would alter TRPV1 currents in MNCs in isotonic solution and whether PKC activation would alter the osmotic activation of ∆N TRPV1‐mediated currents. The mean ramp current traces obtained before and after adding the PKC activator phorbol 12‐myristate 13‐acetate (PMA; 0.2 *μ*mol/L; upper traces) and the digital subtraction of the pre‐ and post‐treatment traces (lower trace) are shown in Figure 9A. The PKC activator caused a significant increase in the mean membrane conductance (0.59 ± 0.08 nS; *n *=* *7 cells) and in the peak ramp current density (2.7 ± 0.3 pA/pF; *n *=* *7 cells), whereas the inactive analog 4*α*‐PMA (0.2 *μ*mol/L) caused no significant increase in mean membrane conductance (0.06 ± 0.07 nS; *n *=* *5 cells) or the peak ramp current density (0.8 ± 0.2 pA/pF; *n *=* *5 cells), as is shown in the left parts of Figure 9C and D (traces not shown). The mean reversal potential of the PKC‐sensitive currents (−34.3 ± 2.3 mV) was not statistically different from the osmotically‐evoked currents suggesting that the PKC activator is increasing ∆N TRPV1‐mediated current.

We tested whether PKC activation would alter the osmotic activation of ∆N TRPV1‐mediated current by treating MNCs with hypertonic saline containing PMA. The mean ramp current traces obtained before and after the switch to the hypertonic saline (upper traces) and the digital subtraction of the pre‐ and post‐treatment traces (lower trace) are shown in Figure 9B. Hypertonic saline in the presence of the PKC activator caused a significantly greater increase in mean membrane conductance (8.60 ± 0.39 nS; *n *=* *5 cells) than it did in the cells in the previous experiments, which were treated with hypertonic saline in the absence of the activator (3.04 ± 0.21 nS; *n *=* *8 cells) and a significantly greater increase in the mean peak ramp current density (29.4 ± 3.9 pA/pF; *n *=* *5 cells) than in the previous experiment (11.9 ± 0.7 pA/pF; *n *=* *8 cells), as is shown in Figure 9C (right) and Figure 9D (right). The mean reversal potential of the osmosensitive current in the presence of the PKC activator was −34.6 ± 2.8 mV and was not statistically different from our previously recorded osmotically‐evoked currents. These data suggest that PKC activation enhances the osmotic activation of ∆N TRPV1‐mediated currents in the MNCs.

## Discussion

We have demonstrated that the MNCs express an osmotically‐evoked Ca^2+^‐dependent PLC pathway that may be important in mediating the electrophysiological changes that MNCs undergo as a consequence of increases in osmolality. Our results show that the magnitude of the osmotically‐evoked decrease in PIP_2_ is consistent with those seen following either receptor‐evoked activation of PLC by Ang II or activation of PLC by a non‐selective PLC activator. The observed time course of the response suggests that the activation occurs in 2 min or less and is rapidly reversible. The time course of the PIP_2_ response is roughly similar to that reported for osmotically‐evoked changes in MNC membrane conductance, which also develop over several tens of seconds (Oliet and Bourque 1993, 1994). Significant changes in PIP_2_ levels were observed with increases of osmolality of at least 10 mosmol kg^−1^, whereas MNCs have been reported to exhibit osmotically‐evoked changes in membrane conductance in response to changes of osmolality of as little 1% (Oliet and Bourque 1994). It is possible however that our method of measuring changes in PIP_2_ underestimates the sensitivity of the response. Our protocol requires comparisons between large populations of treated and untreated MNCs and the variability of measurements of PIP_2_ immunoreactivity (due in part to differences in cell size and shape) may make it difficult to detect small changes in PIP_2_ concentration. Live cell measurements of PIP_2_ might give a better estimate of the sensitivity of the response, but this would require transfection of the MNCs with PIP_2_‐sensitive probes (Lukacs et al. 2013; Borbiro et al. 2015), which is difficult to achieve in acutely isolated adult central neurons (Karra and Dahm 2010).

We next sought to determine the mechanism by which increases in osmolality stimulate PLC and found that the effect depends on activation of TRPV1 channels and the presence of external Ca^2+^. In Figure 3 we presented data showing that the administration of hypertonic solution in the presence of the TRPV1 channel antagonist SB366791 or in the absence of extracellular Ca^2+^ was unable to decrease the PIP_2_ concentration, suggesting that Ca^2+^ influx is necessary for the effect. In Figure 4, we presented data showing that the osmotic activation of PLC is not prevented by the Na^+^ channel antagonist TTX, but is prevented by the presence of L‐type Ca^2+^ channel blocker nifedipine. This suggests that under our conditions acutely isolated rat MNCs do not require action potentials to evoke sufficient Ca^2+^ influx to activate PLC, but do require the influx of Ca^2+^ through L‐type Ca^2+^ channels.

We also treated MNCs with high K^+^ (30 mmol/L) saline, which would be expected to depolarize the MNCs and thereby activate voltage dependent Ca^2+^ channels, and treatment with the Ca^2+^ ionophore A23187 (20 *μ*mol/L), which would directly activate Ca^2+^ influx. Both of these treatments caused a decrease in PIP_2_ levels that was similar to that caused by hypertonic solution (Fig. 5), which suggests that rat MNCs express a Ca^2+^‐dependent PLC pathway that is activated by MNC depolarization and the subsequent Ca^2+^ influx through voltage gated Ca^2+^ channels. The requirement for influx of Ca^2+^ through L‐type Ca^2+^ channels is also true for high K^+^‐induced PLC activation. This effect was blocked by felodipine (30 *μ*mol/L), another L‐type Ca^2+^ channel antagonist (Fig. 5). The high K^+^‐induced decrease in PIP_2_ was also blocked by the presence of a PLC inhibitor (U‐73122; 1 *μ*mol/L) or by the absence of added Ca^2+^ in the external solution.

We did not measure the membrane potential of MNCs exposed to the high K^+^ solutions (although the Goldman Equation suggests that 30 mmol/L K^+^ should depolarize the cells to about ‐40 mV) or the membrane potential of MNCs exposed to +30 mosmol kg^−1^ saline in the presence of TTX, but the results of these experiments imply that action potential firing may not be required to initiate the Ca^2+^ influx that activates PLC under the conditions used. It may therefore be relevant that the MNCs express L‐type Ca^2+^ channels with a low threshold of activation (Fisher and Bourque 1995; Foehring and Armstrong 1996). Whole cell patch clamp studies demonstrated that acutely isolated MNCs express L‐type Ca^2+^ currents with a threshold of activation around −50 mV. These low threshold L‐type Ca^2+^ currents may be mediated by the Ca_V_1.3 subtype (Fisher and Bourque 1995; Foehring and Armstrong 1996), which has been shown to have a lower threshold of activation than the Ca_V_1.2 subtype (Lipscombe et al. 2004). Single cell RT‐PCR of MNCs has shown that both Ca_V_1.2 and Ca_V_1.3 are expressed in MNCs (Glasgow et al. 1999), and immunological experiments have confirmed that Ca_V_1.3 is expressed in MNCs (Fisher et al. 2000; Joux et al. 2001). Under the conditions of our experiments, it may therefore be sufficient to depolarize the MNCs enough to open low threshold L‐type Ca^2+^ channels that do not require action potentials to be activated. This does not eliminate the possibility that influx through both subtypes of L‐type Ca^2+^ channels (or influx of Ca^2+^ through other channels such as the TRPV1 channel; Lukacs et al. 2013; Borbiro et al. 2015) may contribute to the activation of PLC in MNCs in vivo. Prolonged water deprivation has been shown to increase the expression of L‐type Ca^2+^ currents in MNCs (Zhang et al. 2007a) and this could contribute to an increase in the activation of PLC and thereby an enhancement of TRPV1 current when the need for VP release is high.

The activity‐ and Ca^2+^‐dependence of the osmotic activation of PLC has important implications on the identity of the PLC isoform or isoforms that are responsible. Isoforms of PLC have been grouped into 6 major families (PLC *β*, PLC *γ*, PLC *δ*, PLC *ε*, PLC *ζ*, and PLC *η*) based on their sequence homology and domain structure (Suh et al. 2008; Kadamur and Ross 2013). Different subtypes have been shown to be activated by different types of stimuli, including activation of G‐protein coupled receptors (GPCRs) and increases in intracellular Ca^2+^ (Rhee 2001; Suh et al. 2008). The PLC *β* isoforms, for example, are activated by GPCRs, whereas PLC *δ* isoforms are highly Ca^2+^‐dependent. Four different PLC isoforms (PLC *β*4, PLC *δ*1, PLC *δ*4 and PLC *γ*1) have been detected in the SON using DNA microarrays (Hazell et al. 2012). MNCs also express GPCRs such as Ang II (Chakfe and Bourque 2000; Zhang and Bourque 2008; Hazell et al. 2012) and muscarinic acetylcholine receptors (Ghamari‐Langroudi and Bourque 2004; Hazell et al. 2012; Shah et al. 2014) that are linked to the activation of PLC (Rhee 2001; Suh et al. 2008). It is unlikely however that GPCR activation could be involved in the osmotic activation of acutely isolated MNCs, because any signaling molecules released by the cells would rapidly diffuse into the large volume of the bathing solution. The Ca^2+^ dependence of the osmotic activation of PLC suggests the possibility that while the PLC *β*4 isoform may be responsible for the GPCR‐dependent activation of PLC, one or both of the PLC *δ* isoforms may be responsible for the osmotic activation of PLC. Further studies will be required to identify the PLC isoforms underlying these responses.

PLC signaling has been shown to regulate TRPV1‐mediated currents in other cell types (Rohacs et al. 2008) and Ang II has been shown to enhance osmosensory transduction in MNCs through a mechanism that depends on activation of PLC and PKC (Zhang and Bourque 2008). We therefore hypothesized that osmotically‐evoked PLC may contribute to the osmotic activation of ∆N TRPV1 currents in MNCs. We measured ramp currents in acutely isolated rat MNCs using whole cell patch clamp before and after increases in osmolality in the presence and absence of the PLC inhibitor U73122 (1 *μ*mol/L). We observed an osmotically‐evoked current with a reversal potential of about −30 mV (Fig. 6A) and found that this current was diminished, with no change in the reversal potential, in the presence of an inhibitor of PLC. The observed reversal potential is consistent with the reversal potential that has been observed for osmotically‐evoked ramp currents in MNCs (Voisin et al. 1999; Zhang et al. 2007b). MNCs obtained from TRPV1 knockout (*Trpv1* ^−/−^) mice lack both the increase in conductance and the increase in cell firing normally observed in response to increases in osmolality (Sharif Naeini et al. 2006; Zaelzer et al. 2015) and both responses can be rescued by the expression of **∆**N TRPV1, but not wild‐type TRPV1 channels (Zaelzer et al. 2015). These data provide strong evidence that ∆N TRPV1 channels are responsible for mediating the osmotically‐activated current in MNCs. The authors also observed that expressed ∆N TRPV1 channels resulted in a current with a much more positive reversal potential (i.e. close to 0 mV), but this difference appears likely to be caused by unknown factors acting on the ∆N TRPV1 channels under normal physiological conditions in MNCs. Our data suggest that the regulation of osmotically‐evoked currents in MNCs by PLC depends on the modulation of ∆N TRPV1 channels.

The activation of PLC leads to a decrease in PIP_2_ and an increase in IP_3_ and DAG and we next wanted to test which of these changes mediates the enhancement of the TRPV1 current. We tested whether the decrease in PIP_2_ was responsible by comparing current evoked by osmotic stimulation in the presence and absence the PIP_2_ analogue PIP_2_‐diC8 (100 *μ*mol/L) in the patch pipette. We predicted that if the osmotic activation of PLC was enhancing the activation of TRPV1 currents by decreasing PIP_2_, the enhancement should be prevented by the presence of the PIP_2_ analogue and thus the current evoked by the increase in osmolality should be smaller. We found that the response to increased osmolality was not decreased by including the PIP_2_ analogue in the patch pipette (Fig. 7), which suggests that the PLC‐mediated enhancement of osmotically‐evoked TRPV1 currents does not depend on a decrease in PIP_2_.

We next tested whether the PLC‐dependent enhancement of osmotically‐evoked TRPV1 currents depends on the activation of PKC. We show in Figure 8 that the PKC inhibitor GF109203X (2 *μ*mol/L) is able to suppress the osmotic activation of the TRPV1 current whereas an inactive analogue was not (Fig. 8). This suggests that the PLC‐mediated enhancement of the osmotic response depends on the activation of PKC. Lastly, we tested whether direct activation of PKC would enhance TRPV1‐mediated currents in the absence of a change in external osmolality. In Figure 9 we show that treatment with a PKC activator does indeed increase a current with properties consistent with those expected for the TRPV1‐mediated current. Furthermore, treatment with this PKC activator enhances the activation of TRPV1 currents caused by exposure to hypertonic saline. These data support a role for the activation of PLC in the osmotic activation of ∆N TRPV1 currents.

Our data are consistent with earlier results showing that Ang II enhances osmosensory transduction in MNCs through a PLC‐ and PKC‐dependent mechanism (Chakfe and Bourque 2000; Zhang and Bourque 2008). These studies showed that the PKC‐mediated effect of Ang II depends at least in part on an enhancement of cortical actin density (Chakfe and Bourque 2000; Zhang and Bourque 2008) and it remains to be determined whether osmotic activation of PLC mediates changes in cortical actin density in MNCs. Work in other cell types has shown that PKC can enhance the activity of TRPV1 channels by direct phosphorylation at specific sites (Numazaki et al. 2002; Plant et al. 2007). We have not tested whether phosphorylation of TRPV1 channels occurs in MNCs as a result of osmotic activation of PLC and PKC.

MNCs transduce osmotic signals into electrical firing patterns that regulate the body fluid homeostasis by varying the secretion of VP and OT. This transduction depends on the osmotic activation of mechanosensitive TRPV1 channels in the MNCs (Sharif Naeini et al. 2006; Zaelzer et al. 2015) and in other osmosensitive neurons in neighbouring brain regions (Ciura and Bourque 2006; Ciura et al. 2011). Our results demonstrate a novel mechanism for the osmotic regulation of MNC activity. We show that the osmotic activation of PLC, through a Ca^2+^‐dependent mechanism, also contributes to MNC osmosensitivity by enhancing activation of the ∆N TRPV1 currents. We show that this effect is mediated by activation of PKC and is not due to decreased membrane PIP_2_ levels. Our work suggests that osmotic activation of PLC is an important contributor to MNC osmosensitivity and to osmoregulation.

## Conflict of Interest

The authors have no competing interests.
